# Alkyl gallates disrupt *Trypanosoma brucei* lipid droplets

**DOI:** 10.1371/journal.pone.0347099

**Published:** 2026-04-15

**Authors:** Cynthia Mmalebna Amisigo, Gloria Amegatcher, Jack D. Sunter, Theresa Manful Gwira

**Affiliations:** 1 West African Centre for Cell Biology of Infectious Pathogens, University of Ghana, Legon, Accra, Ghana; 2 Department of Biochemistry, Cell and Molecular Biology, University of Ghana, Legon, Accra, Ghana; 3 Department of Biological and Medical Sciences, Oxford Brookes University, Oxford, United Kingdom; Mansoura University Faculty of Veterinary Medicine, EGYPT

## Abstract

African trypanosomiasis is a deadly disease that affects humans and animals, with treatment hindered by drug toxicity and resistance, highlighting the need for alternative therapies. Alkyl gallates, which are plant-derived phenolipids used in the food, cosmetics, and pharmaceutical industries, have been shown to have potent anti-microbial activity; however, their mechanism of action is still unclear. This study investigated the effects of selected alkyl gallates on *Trypanosoma brucei*. The *in vitro* anti-trypanosomal and cytotoxicity of the alkyl gallates were determined using the alamarBlue assay. The effects of the alkyl gallates on lipid droplet dynamics were studied using BODIPY493/503 dye and an endogenously tagged lipid droplet kinase cell line which served as a protein marker of lipid droplets. *In vitro* treatment with octyl and lauryl gallates resulted in rapid cell death with low IC_50_ of 0.7 µM and 0.04 µM respectively. Octyl gallate treatment significantly reduced lipid droplet number within 30 minutes and by 2 hours most cells lacked lipid droplets; however, lipid droplets rebounded once the drug pressure was removed. These results show that alkyl gallates rapidly disrupt lipid droplet homeostasis in *Trypanosoma brucei*. Thus, these gallates could serve as a useful cell biology tool to dissect lipid droplet biogenesis and function in trypanosomes.

## Introduction

*Trypanosoma brucei* (*T. brucei*) is a unicellular flagellate protozoan parasite that causes African trypanosomiasis, an infectious disease affecting humans, wildlife and livestock. Over 55 million individuals are at risk from the disease, and annual losses in livestock production are estimated at $4.5 billion [1,2]. The life cycle of *T. brucei* alternates between the insect vector (tsetse fly) and the mammalian host in which the parasites are found in the extravascular and intravascular compartments including the adipose and the skin [3–5]. Throughout their life cycle, trypanosomes are exposed to different environmental conditions within the mammalian host and the vector such as changes in pH, nutrients and energy sources, and one of the ways they respond to these environmental changes is by adjusting their metabolism [6,7].

Lipid metabolism is an important process in most living organisms and is based on the environment they find themselves [6,8]. For example, trypanosomes found in adipose tissue alter the gene expression of lipid metabolism-related genes to use lipids/fatty acids as a carbon source [5]. Lipid droplets are one of the important components of the lipid metabolism process. They are lipid/fat storing organelles found in the cytosol of all eukaryotes and some prokaryotic cells [9,10]. Apart from serving as storage compartments, lipid droplets are also involved in energy metabolism, lipid trafficking, protection against oxidative stress, and lipotoxicity [11]. Lipid droplets are made up of a monolayer of phospholipid and cholesterol surrounding a hydrophobic core of neutral lipids like triacylglycerides and sterol esters [12]. Studies have shown that the synthesis and/or maintenance of the lipid droplet in *T. brucei* and *Leishmania infantum* requires a protein called the lipid droplet kinase, which localizes to the periphery of the lipid droplet [13,14]. To date, evidence suggests that lipid droplets are essential for the regulation of lipid metabolism in the parasite [15]; however, the tools are lacking to unravel the functional detail of these organelles.

Defining therapeutic targets, unravelling mechanisms of drug resistance and deciphering the mode of action of drugs all rely on a thorough knowledge of the different signaling and metabolic pathways used by these parasites. With regards to lipid metabolism, one way to understand this metabolic process is by interfering with this pathway using inhibitors and one such class of compounds are the lipophilic alkyl gallates [16]. These are plant-derived phenolipids used in the food, cosmetics, and pharmaceutical industries. Alkyl gallates are esters of gallic acid and their structures comprise of a hydrophilic bioactive molecule made up of a catechol moiety and a lipophilic domain comprising of a long chain of carbon atoms (alkyl chain) [17]. These gallates are studied for their antioxidant, anti-microbial, anti-cancer, anti-fungal and anti-protozoan properties [17–21]. Octyl gallate is an inhibitor of desaturases, the enzyme that catalyses the conversion of single to double bonds, especially in the synthesis of unsaturated fatty acids [16,22,23]. These fatty acids are the building blocks for phospholipids, a major component of the lipid droplet monolayer and are also components of triacylglycerides which make up the neutral lipids in the lipid droplets

In this study, we demonstrate that alkyl gallates possess *in vitro* anti-trypanosomal activity and they disrupt lipid droplets and the localization of the associated lipid droplet kinase. Moving forward, this family of compounds will be important for deciphering lipid droplet function.

## Materials and methods

### Cell lines and culture

Bloodstream forms of *T. brucei brucei* GuTat 3.1 and Lister 427 1339 cell lines were cultured in HMI-9 medium supplemented with 10% FCS (Gibco, UK) and β-mercaptoethanol at 37°C in a 5% CO_2_ incubator. The GuTat 3.1 cell lines were used for the IC_50_ determination and growth kinetics and the Lister 427 1339 cell line which contains a construct that encodes a T7 RNA polymerase, a Cas9 nuclease and a TetR was used for reporter cell line generation. Murine macrophages (Raw 264.7 cells) were cultured in DMEM supplemented with 10% FBS, 100X penicillin/streptomycin, 2 g/l NaHCO_3_ and incubated at 37°C with 5% CO_2_. This cell line was used for the cytotoxicity assay. *Trypanosoma brucei brucei,* Strain Lab 110 EATRO was used for the *in vivo* efficacy studies. These cells were sourced from BEI resources, were grown, monitored, and passaged in ICR mice using manufacturer’s protocol.

### Test compounds for *in vitro and in vivo* studies

**Fig 1:**
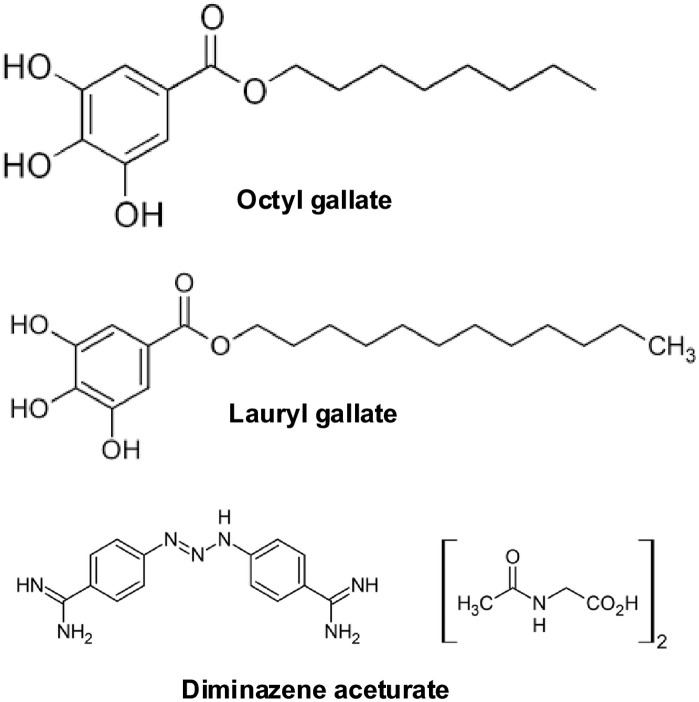
Chemical structures of test compounds. Alkyl gallates (octyl and lauryl gallates) were selected on their lipophilicity and varied alkyl length chains. Diminazene aceturate: standard anti-trypanosomal drug.

All test compounds used (octyl gallate, lauryl gallate and diminazene aceturate) were obtained from Sigma-Aldrich (UK) with diminazene aceturate serving as the positive control. For the *in vitro* studies, stock solutions of the gallates were dissolved in dimethyl sulfoxide (DMSO) and diminazene aceturate was dissolved in water. The compounds were selected based on their lipophilicity and varying alkyl length chain (Fig 1).

### Drug sensitivity test for parental cell lines using alamarBlue assay

Drug sensitivity test of the compounds against bloodstream forms of *T. brucei brucei* was performed using the alamarBlue assay. Compounds were serially diluted in a flat bottom 96 well plate (Costar®) with HMI-9 medium. Trypanosomes were cultured to a density of 1x10^6^ cells/ml and the trypanosome cell suspension was added to the plates to give a final cell density of 4000 cells/ml. The plates were incubated for 72 hours at 37°C in 5% CO_2_. At 5 hours prior to the final incubation, 44 μM of resazurin sodium salt (Sigma-Aldrich, Germany) in phosphate buffered saline was added to each well and the fluorescence measured at 536 nm (excitation) and 588 nm (emission) using the Varioskan Lux Elisa plate reader (ThermoFisher Scientific, USA). Data was analyzed using GraphPad prism software and IC_50_ values (Inhibitory Concentration – 50%) were determined.

### Cytotoxicity against Raw 264.7 macrophage

Toxicity of compounds to Raw 264.7 cells was evaluated. Briefly, the cells were seeded into 96-well plates at ~1x10^5^ cells/ml and incubated for 24 hours for cell adhesion. The cells were treated with varying concentrations of the compounds and incubated for 72 hours at 37°C in 5% CO_2_. At 5 hours prior to the final incubation, 44 μM of resazurin sodium salt (Sigma-Aldrich, Germany) in phosphate buffered saline was added to each well and the fluorescence measured at 536 nm (excitation) and 588 nm (emission) using the Varioskan Lux Elisa plate reader (ThermoFisher Scientific, USA). Cytotoxic Concentration (50%), CC_50_, values were estimated using the Graphpad prism software and the selectivity index (SI) was calculated as the ratio of the CC_50_ to IC_50_ values.

### Analysis of parasite growth

Cells were seeded at an initial density of 1x10^5^ cells/ml, counted and passaged every 24 hours for up to 120 hours in the presence of different drug concentrations, i.e., IC_50_, 2x IC_50_, 5x IC_50_ and 10x IC_50_. A cumulative growth curve was plotted using GraphPad prism software. After analyzing the growth curve and anti-trypanosomal activity, 2x IC_50_ concentrations of octyl gallate (1.4 µM), lauryl gallate (0.08 µM), and diminazene aceturate (0.2 µM) were used for subsequent experiments.

### Morphological and cell cycle analysis

Approximately 1x10^5^ cells/ml were treated with the test compounds (octyl gallate, lauryl gallate, diminazene aceturate) for 6 hours or 12 hours, harvested at 800 g for 5 minutes and washed twice in DMEM. The cells were re-suspended in 1 ml DMEM, 1 µl of Hoechst 33342 (5 µg/ml), harvested and re-suspended in 5–10 µl of DMEM. One microliter of 0.015% paraformaldehyde was added to 2 µl of cell suspension and the total mixture placed on a glass slide with a cover slip. The cells were imaged using the Leica DM5500B microscope with 63x immersion oil objective and a BSI Prime Express camera, controlled by Micromanager 2.0. Counts of the number of kinetoplasts and nuclei present in each cell were performed to determine the cell cycle phenotype.

### Generation of lipid droplet kinase (LDK) reporter cell lines

LDK (Tb427tmp.01.0670) gene was endogenously tagged at the C-terminus by CRISPR-Cas9, as described by [24], to create a reporter cell line. Primers for template and guide PCR were designed using the primer design tool in http://www.leishgedit.net/Home.html. Template plasmids used include pPOTv6-mNeonGreen-blast and pPOTv7-mScarlet-blast. The constructs were generated by long-primer PCR and transfected into cells as described by [25].

### Live cell microscopy of lipid droplet kinase (LDK) and lipid droplet

LDK-tagged and parental cell lines were seeded at a concentration of 1 × 10⁵ cells/ml. The tagged cell lines were exposed to the test compounds for durations of 30 minutes, 1 hour, 2 hours, 4 hours, and 6 hours, whereas the parental cell lines were treated with the test compound for 2 hours. After treatment, cells were harvested at 800 g for 5 minutes and washed twice in DMEM. The LDK-tagged cells were re-suspended in 1 ml DMEM with 1 µl of Hoechst 33342 (5 µg/ml) while parental cells were re-suspended in 1 ml DMEM with 1 µl of Hoechst 33342 and 1 µl of a neutral lipid dye, BODIPY 493/503 (1 mg/ml) for lipid droplet visualisation. Both cell lines were harvested and re-suspended in 5–10 µl of DMEM. One microliter of 0.015% paraformaldehyde was added to 2 µl of cell suspension and the total mixture placed on a glass slide with a cover slip. The cells were imaged using the Leica DM5500B microscope with 63x immersion oil objective and a BSI Prime Express camera, controlled by Micromanager 2.0.

### Alkyl gallate washout and cell recovery

Approximately 1x10^5^ cells/ml of parental and LDK::mNG expressing cells were treated for 2 hours followed by drug washout. After compound treatment the cells were harvested and washed twice in DMEM as previously described and incubated in compound-free media for 30 minutes, 1, 2, 4, and 6 hours for LDK::mNG expressing cells or 2 hours for BODIPY 493/503 stained cells. After incubation, cells were imaged after staining with Hoechst 33342 and fixed with paraformaldehyde.

### Ethical approval and *in vivo* experimental guidelines

Ethical clearance was obtained from the University of Ghana Institutional Animal Care and Use Committee (UG-IACUC) with certificate number (UG-IACUC 006/21–22). Female ICR mice were obtained, bred and housed in the Laboratory of the Animal Facility of the Department of Animal Experimentation-Noguchi Memorial Institute for Medical Research-University of Ghana. All experimental procedures were conducted at this facility adhering to protocol and maintaining quality assurance in accordance with good laboratory practice. The procedures and techniques used were in accordance with the National Institute of Health Guidelines for the Care and Use of Laboratory Animals and established protocols by the Organization for Economic Co-operation and Development (OECD). The mice were euthanized by inhalation of isoflurane and cervical dislocation, and all efforts were made to minimize suffering. Mice were observed at least twice a day for signs of distress/pain, including significant weight loss, loss of motility or decreased activity, irregular breathing, discharge around eyes, patches or loss of fur. None of the mice showed any signs of pain during the observation period. No painkillers were used. All observations and procedures were done by trained personnel at the Department of Animal Experimentation-Noguchi Memorial Institute for Medical Research-University of Ghana.

### Efficacy studies

Mice were maintained in standard ventilated rodent cages containing sterilized softwood shavings. All animals had *ad libitum* access to rodent feed pellets and clean drinking water. Housing conditions were strictly regulated, with a temperature of 23 ± 2 °C, relative humidity maintained at 60–70%, and a 12-hour light/dark cycle. Animals were kept in their cages and allowed to acclimatize to the laboratory conditions for 3 days before the start of the experiment.

A total of 25 female ICR mice were used for the efficacy studies and the weight of the mice ranged between 25–30 g. The mice were grouped in 5 with 5 mice per group. Group 1: Untreated & Uninfected; Group 2: Infected & Untreated; Group 3: Diminazene aceturate treated group (DA 3.5 mg/kg); Group 4: Octyl gallate treated group (OG 62.5 mg/kg); Group 5: Octyl gallate treated group (OG 1000 mg/kg). The mice in groups 2–5 were infected intraperitoneally (i.p.) with 50 µl of blood containing *T. brucei* at 5x10^4^ parasites and the animals were then treated at the peak of parasitemia. Mice in Group 3 were treated once a day with 3.5 mg/kg diminazene aceturate via i.p. while those in Groups 4 and 5 were orally dosed once a day with 62.5 mg/kg and 1000 mg/kg of octyl gallate respectively. The untreated & infected group were orally dosed with the vehicle (Tragacanth solution). The ProTox-II database was used to get a predicted LD_50_ value (50% lethal dose) of octyl gallate which was approximately 2000 mg/kg. The first (1000 mg/kg) and fifth concentrations (62.5 mg/kg) of a two-fold serial dilution of the lethal dose were used as the treatment concentrations. The parasitemia was checked daily by microscopic examination of tail blood using Giemsa stain. Mice with cleared parasitemia were left without treatment for 3 days to monitor for relapse after which they were immediately euthanized. The untreated mice were also euthanized at the end of the 8- day study period. Complete elimination of blood parasitemia, lack of relapse and survival of mice were used as markers for treatment efficacy.

### Statistical analysis

Results were expressed as the means ±SD using Unpaired Student#39;s t test and Welch’s t-test comparing the untreated and treated groups. Data was analysed using the GraphPad prism and p-value of <0.05 was considered significant. The p-values for all experimental data are presented in S1 Table.

## Results

### Alkyl gallates possess anti-trypanosomal activity *in vitro* but not *in vivo*

**Fig 2:**
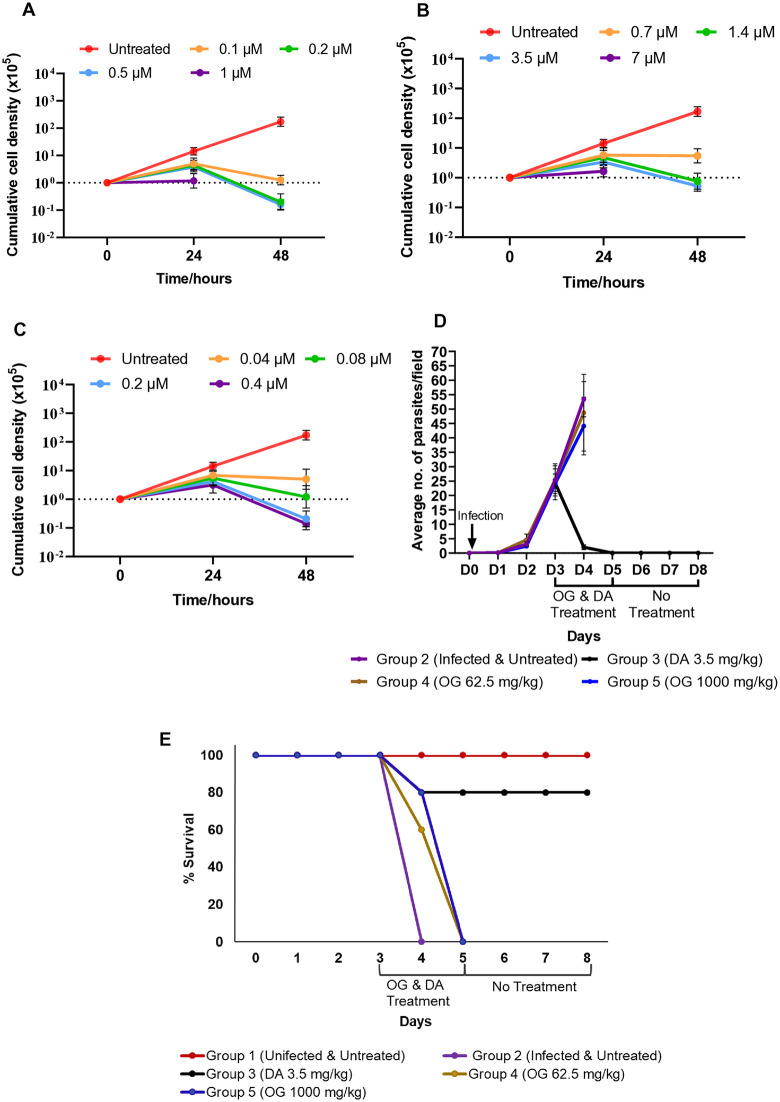
Alkyl gallates possess anti-trypanosomal activity *in vitro* but not *in vivo.* Cumulative growth curve of trypanosomes treated with **(A)** Diminazene aceturate **(B)** Octyl gallate and **(C)** Lauryl gallate at IC_50_, 2x IC_50_, 5x IC_50_ and 10x IC_50_. Error bars for the growth curve show the standard deviation of the mean of three technical replicates **(D)** Blood parasitemia of *T. brucei* infected mice. The parasitemia was determined by counting at least 30 Giemsa-stained parasites per field at a 100x magnification. **(E)** Survival of *T. brucei* infected mice during efficacy studies. Sample size: Group 1: Days 1-8 (n = 5), Group 2: Days 0-3 (n = 5), Day 4 (n = 0); Group 3: Days 0-3 (n = 5), Days 4-8 (n = 4), Group 4: Days 0-3 (n = 5), Day 4 (n = 3), Day 5 (n = 0), Group 5: Day 0-3 (n = 5), Day 4 (n = 4), Day 5 (n = 0). Error bars for blood parasitemia represent the standard deviation of the mean of values within each group.

We first sought to investigate the *in vitro* activity of alkyl gallates on *Trypanosoma brucei* bloodstream forms. Diminazene aceturate (standard anti-trypanosomal drug) exhibited anti-trypanosomal activity, with IC_50_ of 0.1 µM (Table 1) resulting in a 70% decrease in cell density at 2x IC_50_ after 24 hours (Fig 2A and S2 Table). Octyl and lauryl gallate also showed anti-trypanosomal activity, with IC_50_ values of 0.7 µM and 0.04 µM, respectively (Table 1) and a corresponding 72% and 74% decrease in cell density after 24 hours at 2x IC_50_ (Fig 2B and 2C, and S2 Table). The alkyl gallates had low toxicity to murine raw macrophages compared to the standard animal drug, diminazene aceturate, resulting in a high selectivity index of >100 that indicates the compounds were non-toxic to the host cell but selective for the parasite. (Table 1).

**Table 1:** Anti-trypanosomal and cytotoxic activity of alkyl gallates.

Test compounds	IC_50_(µM)±SD	CC_50_(µM)±SD	SI (CC_50_ / IC_50_)
**Diminazene Aceturate**	0.1 ± 0.02	0.05 ± 0.05	0.5
**Octyl Gallate**	0.7 ± 0.06	110.0 ± 0.02	>100^a^
**Lauryl Gallate**	0.04 ± 0.002	32.0 ± 0.02	>100^a^

^a^SI > 10, means the compounds are selectively toxic to trypanosomes

Given the anti-trypanosomal effects of the alkyl gallates *in vitro*, we next sought to investigate whether they could cure a trypanosome infection in mice. The mice were infected with trypanosomes and then treated with either octyl gallate or diminazene aceturate from day 3–5 post infection (Fig 2D and S2 Table). Parasitemia increased from day 1–3 in all groups and continued to increase in all groups except the diminazene aceturate treated mice, which showed a sharp decrease in parasitemia by day 4 and on day 5 no parasites were detected (Fig 2D and S2 Table). Even after cessation of diminazene aceturate treatment, there was no relapse and 80% of the animals survived until completion of the experiment (Fig 2E and S2 Table). Mice treated with both concentrations of octyl gallate showed an increase in parasitemia treatment until day 4 at a similar rate to the untreated animals (Fig 2D and S2 Table) with a 0% survival rate by day 5 (Fig 2E and S2 Table). The data show that diminazene aceturate as expected was successful in clearing blood parasitemia but octyl gallate was unable to clear trypanosomes from the mice.

### Alkyl gallate treatment causes minimal effect on cell cycle but adversely affects cell morphology

**Fig 3:**
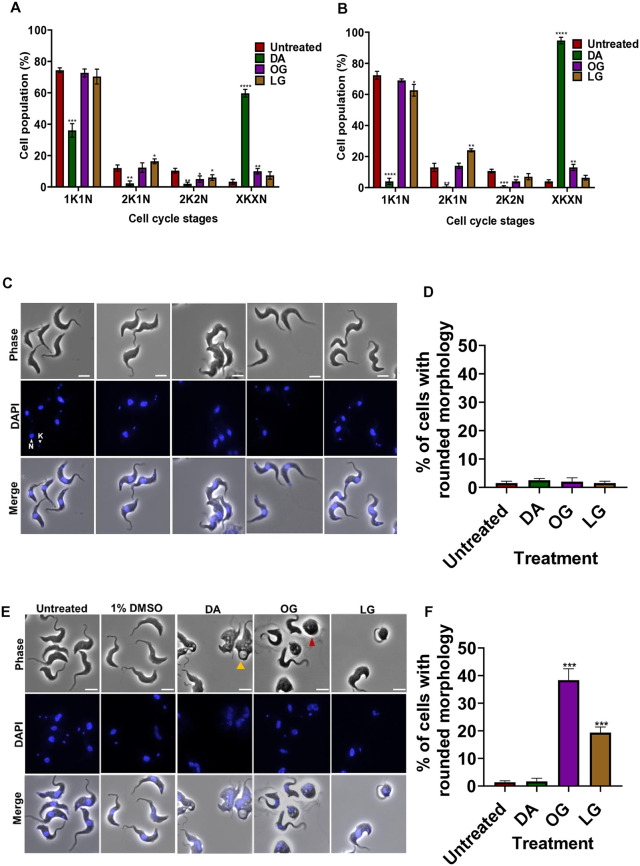
Alkyl gallates cause rounding of cells with minimal effect on the cell cycle. Analysis of cell cycle stages after (A) 6 hours of treatment and (B) 12 hours of treatment. (C) Representative images of cell morphology after 6 hours of treatment. (D) Percentage of cells with rounded morphology after 6 hours of treatment. (E) Representative images of cell morphology after 12 hours of treatment. (F) Percentage of cells with rounded morphology after 12 hours of treatment. Treated cells were stained with Hoechst 33342 and imaged. The nucleus is indicated as N and the kinetoplast as K, yellow and red arrows show the enlarged flagellar pocket and rounded cells respectively. Error bars show standard deviation of the mean of 3 technical replicates (n = 200 cells per experiment). ****p  <  0.0001; ***p  <  0.001; **p  <  0.01; *p  <  0.05 by Unpaired Student#39;s t test. Scale bar = 5 μm. DA = Diminazene aceturate, OG = Octyl gallate, LG = Lauryl gallate, Untreated = 1% DMSO.

We hypothesized that since these compounds are lipophilic and are structurally different to other anti-trypanosomal therapeutics, they would have a novel mode of action and could be re-purposed as a tool to interrogate trypanosome cell biology. To investigate the mode of action of octyl and lauryl gallate, we initially evaluated their effect on the cell cycle of the parasite. The cells were incubated with 2x IC_50_ concentrations of the compounds, stained with the DNA stain Hoechst, and imaged by fluorescence microscopy. The pattern of kinetoplast and nucleus duplication and segregation in trypanosomes occurs in a regulated and consistent manner. At the start of the cell cycle, the cell has 1 kinetoplast (K) and 1 nucleus (N), after which the kinetoplast divides to give a 2K1N cell, next the nucleus divides to give a 2K2N cell before cytokinesis occurs. After diminazene aceturate treatment there was a significant effect on the cell cycle, characterized by a decrease in the proportion of the 1K1N, 2K1N and 2K2N cells and an increase in abnormal cells that do not fall within the normal KN annotation (xKxN) relative to the untreated control (Fig 3A and 3B, and S2 Table). In comparison, octyl gallate had little effect on the cell cycle with a decrease in 2K2N cells and an increase in xKxN cells after 6 and 12 hours of treatment (Fig 3A and 3B, and S2 Table). Lauryl gallate also had a minor effect on the cell cycle indicated by an increase in proportions of the 2K1N cells a decrease in the 2K2N cells after 6 hours as well as a decrease in 1K1N cells and an increase in 2K1N cells after 12 hours (Fig 3A and 3B, and S2 Table). Overall, diminazene aceturate inhibited kinetoplast duplication and cell division as expected, while both octyl and lauryl gallate had minimal effect on the cell cycle. This suggests that the alkyl gallates do not affect duplication and segregation of these organelles.

Next, we examined the changes in cell morphology after treatment. After 6 hours of treatment with all the compounds, the cells had a normal morphology, characterized by a long, slender body and an attached flagellum with less than 5% of cells exhibiting cell rounding (Fig 3C and 3D, S3 Fig, and S2 Table). However, after 12 hours of treatment, ~ 40% and ~20% of octyl and lauryl gallate treated cells had started to round, respectively (Fig 3E and 3F, S4 Fig, and S2 Table). In addition, cells treated with diminazene aceturate for 12 hours also had an aberrant morphology, with an enlarged flagellar pocket and distorted cell shape; however, only ~3% had rounded in a similar manner as the cells treated with alkyl gallates (Fig 3E and 3F, S4 Fig, and S2 Table). This shows that octyl and lauryl gallate cause a distinct change to the morphology of the parasite.

### Alkyl gallates disrupt lipid droplets

**Fig 4:**
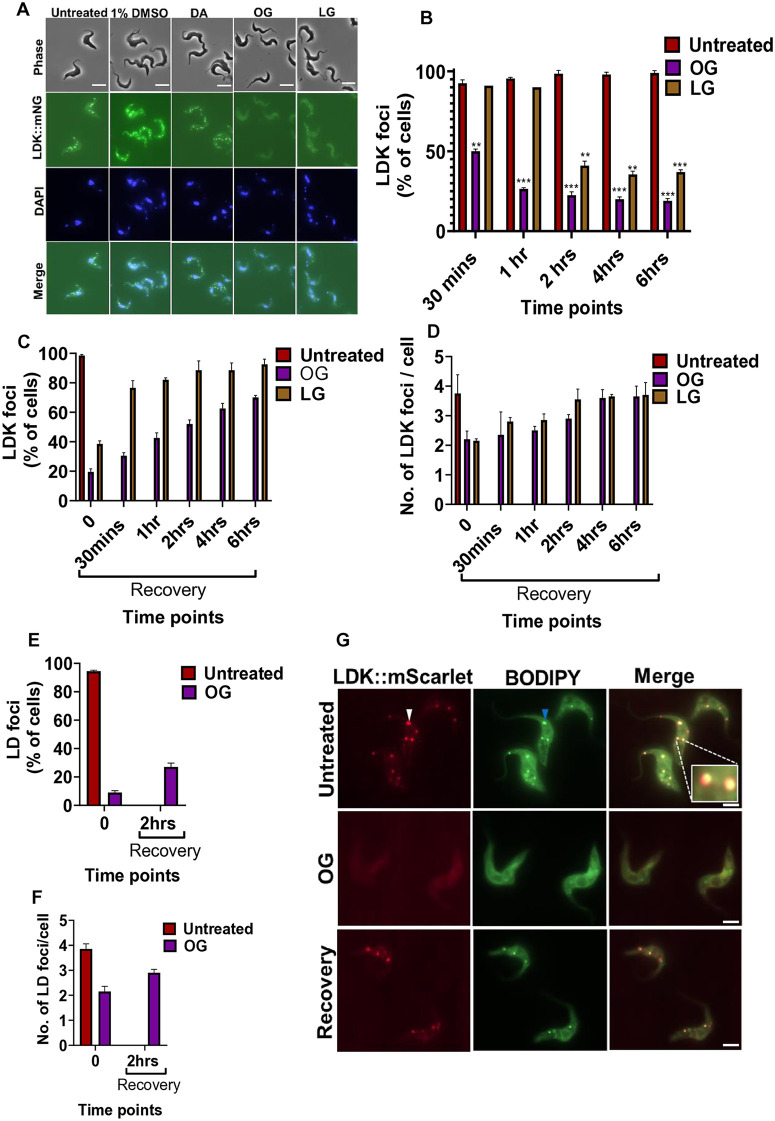
Disruption and recovery of lipid droplet kinase (LDK) and lipid droplet (LD) foci by alkyl gallate treatment. (A) Representative images of LDK tagged cells after 6 hours of compound treatment. (B) Percentage of cells with LDK foci after 30 minutes, 1, 2, 4, and 6 hours of treatment. (C) Percentage of cells with LDK foci after alkyl gallate treatment and recovery. (D) Number of LDK foci per cell following alkyl gallate treatment and recovery. (E) Percentage of cells with LD after 2 hours of octyl gallate treatment and recovery. (F) Number of LD per cell after 2 hours of octyl gallate treatment and recovery. (G) Co-localization of LDK and LD following octyl gallate treatment and recovery at 2 hours. Cells were stained for lipid droplet using BODIPY493/503. LDK foci (white arrow head), lipid droplet (blue arrowhead). Scale bar 5 µm, insert: 2 µm. Error bars show the standard deviation of the means of 2 technical replicates (n = 100 cells per time point). ****p  <  0.0001; ***p  <  0.001; **p  <  0.01; *p <  0.05 by Unpaired Student#39;s t test with Welch’s correction (Welch’s t test). DA = Diminazene aceturate, OG = Octyl gallate, LG = Lauryl gallate, Untreated = 1% DMSO. NB Time point 0 = 2 hours after treatment.

Given the lipophilic nature of the alkyl gallates and their effect on cell morphology and reported effect on fatty acid synthesis [16], we hypothesized these compounds affect lipid metabolism/homeostasis. An important organelle in lipid homeostasis are lipid droplets; therefore, to analyze the effect of alkyl gallate treatment on lipid droplets, we generated a cell line expressing lipid droplet kinase fused to mNeonGreen (mNG). We incubated these cells with diminazene aceturate, octyl and lauryl gallate before quantifying the number of cells with LDK::mNG foci after 30 minutes, 1, 2, 4, and 6 hours of treatment (Fig 4A and 4B, S5 Fig, and S2 Table). After 30 minutes of treatment, about 50% of octyl gallate treated cells had LDK foci and this decreased to about 20% and 40% by 2 hours for octyl and lauryl gallate respectively and this level was maintained for the rest of the experiment (Fig 4A and 4B, S5 Fig, and S2 Table). This shows that alkyl gallates disrupt lipid droplet kinase localization.

Next, we determined whether the LDK foci would recover once the drug pressure was removed. The LDK::mNG expressing cells were treated with octyl and lauryl gallates for 2 hours, then washed and re-suspended in alkyl gallate free media and imaged at different time points. After 2 hours of treatment, there was a decrease in the portion of cells containing LDK::mNG foci (Fig 4C and S2 Table). After washout and recovery, the number of cells containing LDK::mNG foci increased steadily (Fig 4C and S2 Table). In addition, we examined the number of LDK::mNG foci present in those cells, which retained LDK::mNG foci even after treatment. After 2 hours of treatment those cells, in which LDK::mNG foci were retained had ~ 2 LDK::mNG foci in comparison to 3–4 foci without treatment and the number of LDK::mNG foci slowly increased after the treatment was washed out in line with the recovery of cells with LDK::mNG foci (Fig 4D and S2 Table). This shows that the effect of alkyl gallates on treatment on LDK::mNG was reversible after short periods of treatment.

Next, we proceeded to determine the effect of octyl gallate on the lipid components of the lipid droplet, using the neutral lipid stain BODIPY493/503 (Fig 4E and S2 Table). We focused on octyl gallate as it had a more rapid effect on LDK localization. After 2 hours of treatment with octyl gallate, about 90% of the parental cells did not have lipid droplets and when octyl gallate was washed out, the number of lipid droplets began to recover with ~27% of cells seen with observable lipid droplets after 2 hours (Fig 4E and S2 Table). In addition, we examined the number of lipid droplets present in those cells, which retained lipid droplets even after treatment. After 2 hours of treatment with octyl gallate those cells, in which lipid droplets were retained had ~ 2 lipid droplets in comparison to ~4 lipid droplets without treatment (Fig 4F and S2 Table). The number of lipid droplets increased after the treatment was washed out in line with the recovery of cells with lipid droplets. This shows that octyl gallate treatment also effects the localization of lipids to lipid droplets, in addition to LDK and that this effect was reversible.

Next, we sought to confirm both LDK and lipids were simultaneously lost from lipid droplets. We generated a cell line in which LDK was endogenously tagged with mScarlet. We then treated this cell line with octyl gallate for 2 hours before washing out the compound and letting the cells recover for 2 hours and then stained them with BODIPY493/503 (Fig 4G and S6 Fig). Without treatment the LDK::mScarlet overlapped with the BODIPY493/503 signal from the lipid droplets and after 2 hours of treatment with octyl gallate both the LDK::mScarlet and lipid droplet signal were lost from most cells (Fig 4G and S6 Fig). After washout, foci of both LDK::mScarlet and lipid droplet recovered (Fig 4G and S6 Fig). This suggests that the effect of octyl gallate on LDK and lipids occurs on a similar timescale.

## Discussion

Octyl and lauryl gallates are plant-derived phenolipids used in the food, cosmetics, and pharmaceutical industries [26–28]. They are lipid derivatives of gallic acid within the alkyl gallate class and have been shown to have potent anti-microbial activity [18,19]. In this study, treatment with octyl and lauryl gallate resulted in a dose-dependent inhibitory effect on trypanosomes, with IC_50_ of 0.7 µM and 0.04 µM respectively. Both compounds showed low toxicity to murine macrophages and a high selectivity index (>100). These findings suggest that the alkyl gallates possess good anti-trypanosomal activities, parasite-specific selectivity and could serve as promising therapeutic candidates [29]. These toxicity findings are also consistent with other studies showing that alkyl gallates are less toxic to normal human cells while being relatively non-genotoxic and non-mutagenic, further supporting their therapeutic potential [21,30]. Though both octyl and lauryl gallate exhibited good *in vitro* activity, octyl gallate showed no efficacy in trypanosome-infected mice.

Treatment of the cells with either octyl or lauryl gallate caused cell rounding after 12 hours but there was limited effect on the cell cycle. This phenotype was distinct to that of one of the standard anti-trypanosomal drugs, diminazene aceturate which caused an abnormal cell morphology, with an enlarged flagellar pocket and a significant defect in the cell cycle, with the accumulation of xKxN cells. This phenotype of the diminazene aceturate treated cells was expected given that the compound has been shown to cause a loss in the kinetoplast DNA by binding to its minor grooves at AT-rich sites, inhibiting the mitochondrial type II topoisomerase and DNA replication [31–33]. The exact mechanism of action of the alkyl gallates is unclear. Studies suggest that the activity of alkyl gallate is associated with their lipophilicity, which increases the permeability of cell membranes and enables the compounds to be readily taken up by the cell [34,35]. In bacteria, octyl gallate exhibits its anti-bacterial property by interacting with the nuclear content to alter gene replication after 12 hours of treatment [36], while in cancer, octyl gallate is thought to inhibit cell cycle progression by altering the expression of key cell cycle regulatory proteins [21]. In trypanosomes, the changes in cell morphology and cell cycle after alkyl gallate treatment were only observed after extended treatment times, suggesting that these could be the subsequent actions of these compounds resulting from downstream events leading to cell death.

In contrast to the slow onset of the cell morphological changes after alkyl gallate treatment, we saw a rapid effect on lipid droplets, in which most of the cells lost their lipid droplets after 2 hours of treatment. This loss was seen for both the lipid components and the protein, LDK, which localizes to the periphery of the lipid droplet. The rapid disruption of the lipid droplets on alkyl gallate treatment suggest this is the likely the initial site of action of these compounds in *T. brucei*. Interestingly, this is almost the opposite effect seen in other systems, where octyl gallate has been shown to induce lipid droplet accumulation in hepatocellular carcinoma cell line and cause the upregulation of lipid metabolism gene [37].

The importance of lipid droplets for parasite survival is not clear. The depletion of LDK in trypanosomes resulted in a loss of lipid droplets, yet there was no effect on growth [13]. However, in trypanosomes the enzyme lipin is essential and its depletion caused a reduction in lipid droplets, though there were additional deleterious changes to the mitochondria seen, which might explain the essentiality of this enzyme [15]. In the closely related parasite, *Leishmania infantum*, LDK could be deleted and so is dispensible for cell growth in culture. Yet, deletion of LDK did result in a significant reduction of lipid droplets during parasite stationary phase, accompanied by reduced macrophage infectivity [14]. Together, this suggests that lipid droplet disruption after treatment with alkyl gallates is not the primary cause of trypanosome cell death. Instead, we propose that prolonged alkyl gallate treatment causes a range of lipid-related metabolic stress resulting in cytotoxicity and cell death, with the disruption of lipid droplets being one of the early phenotypes of this stress. Interestingly, the loss of lipid droplets seen after two hours of alkyl treatment is reversible, if the compound is washed out. This effect may point to plasticity in parasite lipid metabolism, which enables lipid droplet restoration or that after only a short treatment period the disruption to their lipid metabolism is not severe. Future studies should focus on defining whether there is a loss of lipid droplet reversibility after extended periods of alkyl gallate treatment.

**Fig 5:**
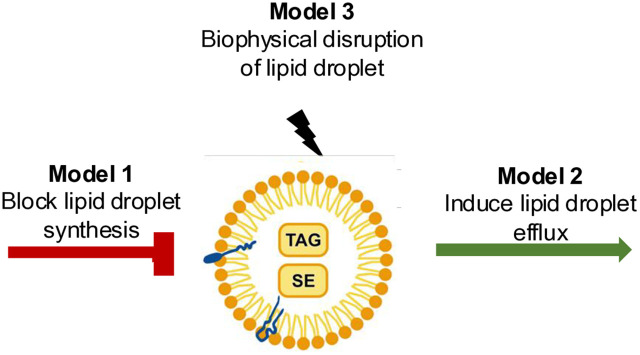
Proposed models for the mechanism of action of alkyl gallate in *T. brucei.* Model 1: Alkyl gallate could interfere with the lipid metabolic pathways, impairing lipid droplet formation leading to droplet loss. Model 2: Alkyl gallate could interfere with the lipid metabolism pathway, forcing cells to release the lipid droplet content leading to droplet loss. Model 3: Alkyl gallate could interfere with the droplet’s physical structure leading to the collapse of the droplet. TAG = Triacylglycerol; SE = Sterol esters.

It is still uncertain how these alkyl gallates cause lipid droplet disruption in trypanosomes. The presence of lipid droplets will rely on a balance between the synthesis of the lipids found in droplets and their utilization by the cell. Based on our findings and previous studies, we propose three models as to how these compounds could be acting to cause a loss of lipid droplets (Fig 5). The first model is a block in lipid droplet biogenesis/synthesis, which is supported by previous work that showed octyl gallate acts as an inhibitor of desaturases [16]. This inhibition may affect the biosynthesis of triacylglycerides thus leading to lipid droplet loss, if there is continued efflux from the droplets for cellular metabolism (Fig 5). The second model is an induction in lipid droplet efflux. Here, the alkyl gallate could interfere with the lipid metabolism pathway, forcing the cell to mobilize the lipid droplets releasing their contents (Fig 5). Finally, in the third model the alkyl gallates may interact with the lipid droplets directly causing a biophysical disruption that leads to droplet loss (Fig 5). This latter model is attractive based on the rapid loss of lipid droplets on alkyl gallate treatment and their recovery after washout; however, further work would be required to define the mode of action of these compounds.

Overall, this study shows that alkyl gallates possess *in vitro* anti-trypanosomal activity and provides novel insights into their mode of action in *T. brucei.* While our findings clearly demonstrate lipid droplet disruption after alkyl gallate treatment in *T. brucei*, the molecular targets of alkyl gallates are yet to be identified. To gain deeper insight into how these compounds affect lipid metabolism, future work should include lipidomic profiling to provide an overview of cellular lipid composition after alkyl gallate treatment, complemented with molecular and biochemical studies. Together, these approaches will help to confirm the proposed mechanisms and to fully elucidate the role(s) of alkyl gallates in modulating lipid homeostasis in trypanosomes.

## Conclusions

The study provides insight into the mode of action of alkyl gallates in *Trypanosoma brucei,* with the compounds primarily interfering with lipid droplets. Moreover, considering the importance of lipid metabolism and the limited information regarding its role in trypanosome’s survival, compounds that target lipid metabolism could not only serve as effective anti-trypanosomal agents but also as promising tools for elucidating the roles of lipids in these parasites.

## Supporting information

S1 Table Statistical tests with p-values from experimental data. (PDF)

S2 Table Minimal data set. (XLSX)

S3 Fig Representative images. (PPTX)

S4 Fig Representative images. (PPTX)

S5 Fig Representative images. (PPTX)

S6 Fig Representative images. (PPTX)
