# Supplementary material for: Alkyl gallates disrupt Trypanosoma brucei lipid droplets
Source: PLoS One. 2026 Apr 15;21(4):e0347099. doi: 10.1371/journal.pone.0347099 (PMC13082637; doi:10.1371/journal.pone.0347099)
Supplement: S1 Table — (PDF) [file pone.0347099.s001.pdf]

**S1 Table. Statistical tests with p-values from experimental data**

| Figure title | Statistical Test | p-value | Asterisks |
|---|---|---|---|
| **Fig 3A: Cell cycle analysis after 6 hours of treatment** | | | |
| UT vrs DA (1K1N) | Unpaired Student's t test | 0.0001 | *** |
| UT vrs OG (1K1N) | Unpaired Student's t test | 0.2099 | |
| UT vrs LG (1K1N) | Unpaired Student's t test | 0.1696 | |
| UT vrs DA (2K1N) | Unpaired Student's t test | 0.0027 | ** |
| UT vrs OG (2K1N) | Unpaired Student's t test | 0.8820 | |
| UT vrs LG (2K1N) | Unpaired Student's t test | 0.0406 | * |
| UT vrs DA (2K2N) | Unpaired Student's t test | 0.0014 | ** |
| UT vrs OG (2K2N) | Unpaired Student's t test | 0.0214 | * |
| UT vrs LG (2K2N) | Unpaired Student's t test | 0.0314 | * |
| UT vrs DA (XKXN) | Unpaired Student's t test | <0.0001 | **** |
| UT vrs OG (XKXN) | Unpaired Student's t test | 0.0075 | ** |
| UT vrs LG (XKXN) | Unpaired Student's t test | 0.0666 | |
| **Fig 3B: Cell cycle analysis after 12 hours of treatment** | | | |
| UT vrs DA (1K1N) | Unpaired Student's t test | <0.0001 | **** |
| UT vrs OG (1K1N) | Unpaired Student's t test | 0.1000 | |
| UT vrs LG (1K1N) | Unpaired Student's t test | 0.0211 | * |
| UT vrs DA (2K1N) | Unpaired Student's t test | 0.0018 | ** |
| UT vrs OG (2K1N) | Unpaired Student's t test | 0.6130 | |
| UT vrs LG (2K1N) | Unpaired Student's t test | 0.0025 | ** |
| UT vrs DA (2K2N) | Unpaired Student's t test | 0.0002 | *** |
| UT vrs OG (2K2N) | Unpaired Student's t test | 0.0016 | ** |
| UT vrs LG (2K2N) | Unpaired Student's t test | 0.0514 | |
| UT vrs DA (XKXN) | Unpaired Student's t test | <0.0001 | **** |
| UT vrs OG (XKXN) | Unpaired Student's t test | 0.0022 | ** |
| UT vrs LG (XKXN) | Unpaired Student's t test | 0.0913 | |
| **Fig 3D: Percentage of cells with rounded morphology after 6 hours of treatment** | | | |
| UT vrs DA | Unpaired Student's t test | 0.3739 | |
| UT vrs OG | Unpaired Student's t test | 0.3739 | |
| UT vrs LG | Unpaired Student's t test | 0.5185 | |
| **Fig 3F: Percentage of cells with rounded morphology after 12 hours of treatment** | | | |
| UT vrs DA | Unpaired Student's t test | 0.6779 | |
| UT vrs OG | Unpaired Student's t test | 0.0001 | *** |

| Figure title | Statistical Test | p-value | Stars |
|---|---|---|---|
| **Fig 3F: Percentage of cells with rounded morphology after 12 hours of treatment** | | | |
| UT vrs LG | Unpaired Student's t test | 0.0001 | *** |
| **Fig 4B: Percentage of cells with LDK foci after 30 minutes, 1, 2, 4, and 6 hours of treatment** | | | |
| UT vrs OG (30 mins) | Welch's t test | 0.0035 | ** |
| UT vrs LG (30 mins) | Welch's t test | 0.5000 | |
| UT vrs OG (1 hr) | Welch's t test | 0.0001 | *** |
| UT vrs LG (1 hour) | Welch's t test | 0.0577 | |
| UT vrs OG (2 hours) | Welch's t test | 0.0008 | *** |
| UT vrs LG (2 hours) | Welch's t test | 0.0027 | ** |
| UT vrs OG (4 hours) | Welch's t test | 0.0003 | *** |
| UT vrs LG (4 hours) | Welch's t test | 0.0018 | ** |
| UT vrs OG (6 hours) | Welch's t test | 0.0003 | *** |
| UT vrs LG (6 hours) | Welch's t test | 0.0005 | *** |

****$p < .0001$; ***$p < .001$; **$p < .01$; *$p < .05$. DA = Diminazene aceturate, OG = Octyl gallate, LG = Lauryl gallate, Untreated = UT
