# Supplementary material for: Alkyl gallates disrupt Trypanosoma brucei lipid droplets
Source: PLoS One. 2026 Apr 15;21(4):e0347099. doi: 10.1371/journal.pone.0347099 (PMC13082637; doi:10.1371/journal.pone.0347099)
Supplement: S4 Fig — (PPTX) [file pone.0347099.s004.pptx]

## Slide 1
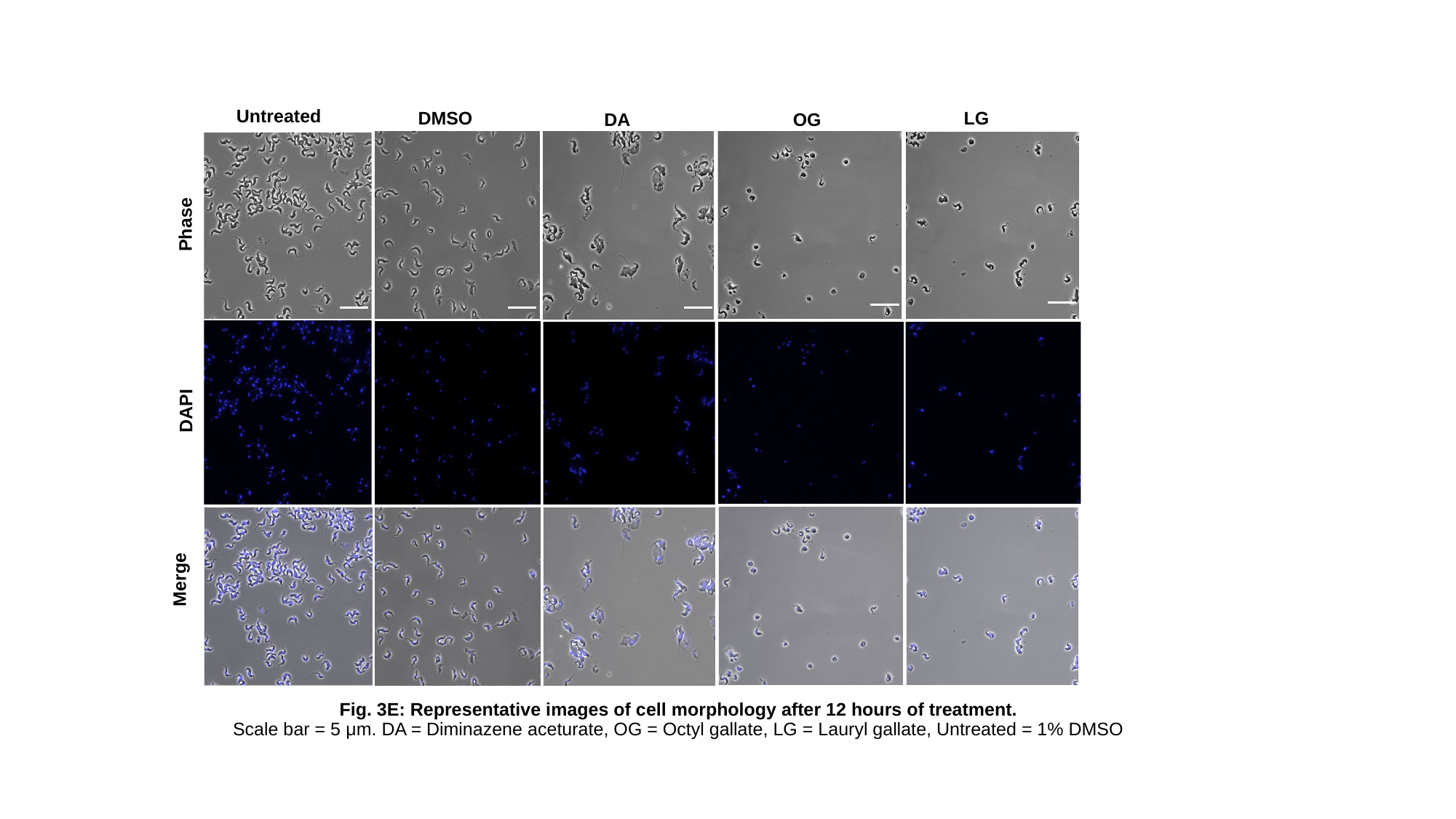

Untreated
DMSO
LG
OG
DA
Phase
DAPI
Merge
# Fig. 3E: Representative images of cell morphology after 12 hours of treatment. Scale bar = 5 μm. DA = Diminazene aceturate, OG = Octyl gallate, LG = Lauryl gallate, Untreated = 1% DMSO
