# Supplementary material for: Alkyl gallates disrupt Trypanosoma brucei lipid droplets
Source: PLoS One. 2026 Apr 15;21(4):e0347099. doi: 10.1371/journal.pone.0347099 (PMC13082637; doi:10.1371/journal.pone.0347099)
Supplement: S5 Fig — (PPTX) [file pone.0347099.s005.pptx]

## Slide 1
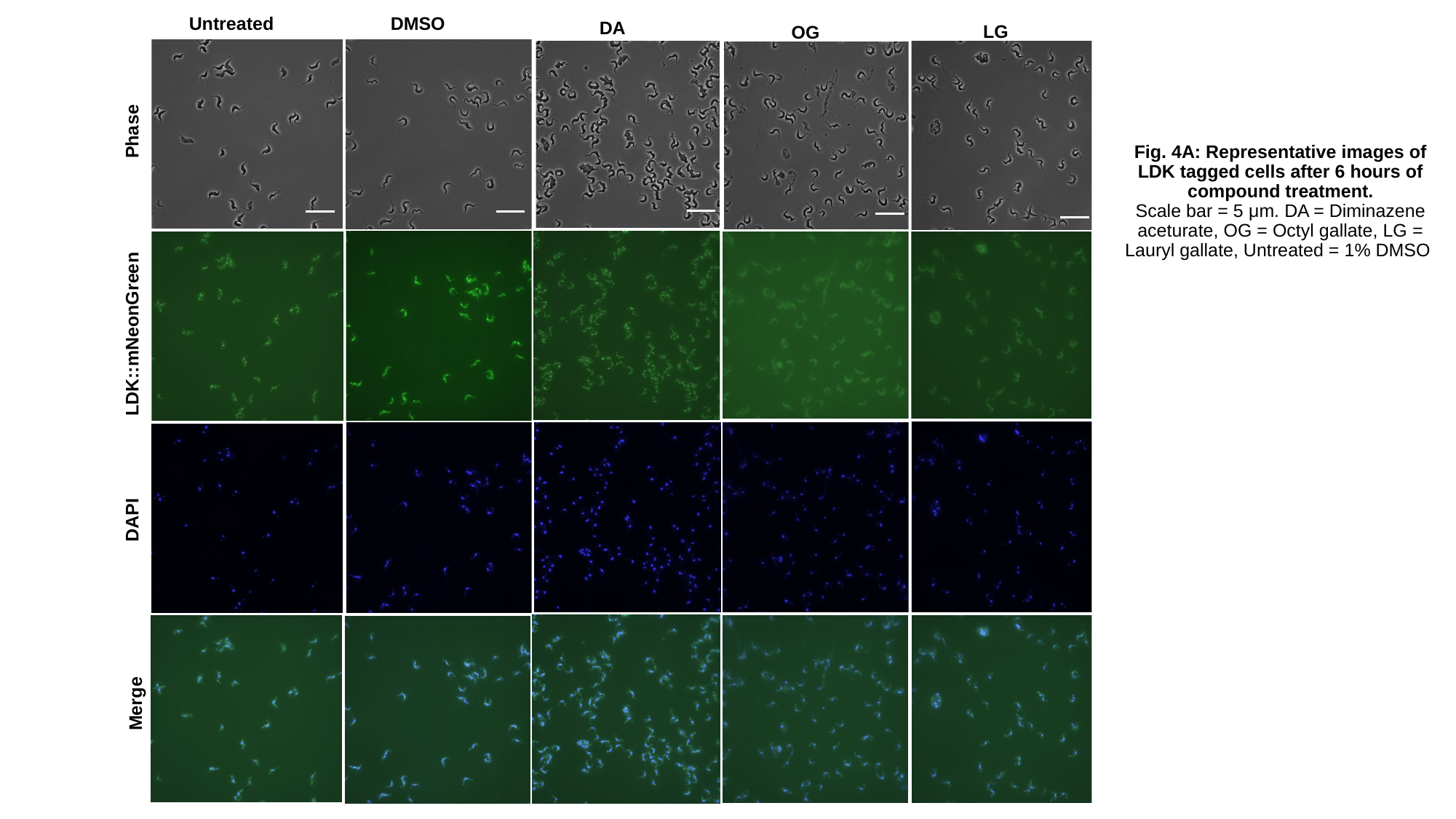

Untreated
DMSO
DA
LG
OG
Phase
LDK::mNeonGreen
DAPI
Merge
# Fig. 4A: Representative images of LDK tagged cells after 6 hours of compound treatment. Scale bar = 5 μm. DA = Diminazene aceturate, OG = Octyl gallate, LG = Lauryl gallate, Untreated = 1% DMSO
