# Supplementary material for: Alkyl gallates disrupt Trypanosoma brucei lipid droplets
Source: PLoS One. 2026 Apr 15;21(4):e0347099. doi: 10.1371/journal.pone.0347099 (PMC13082637; doi:10.1371/journal.pone.0347099)
Supplement: S6 Fig — (PPTX) [file pone.0347099.s006.pptx]

## Slide 1
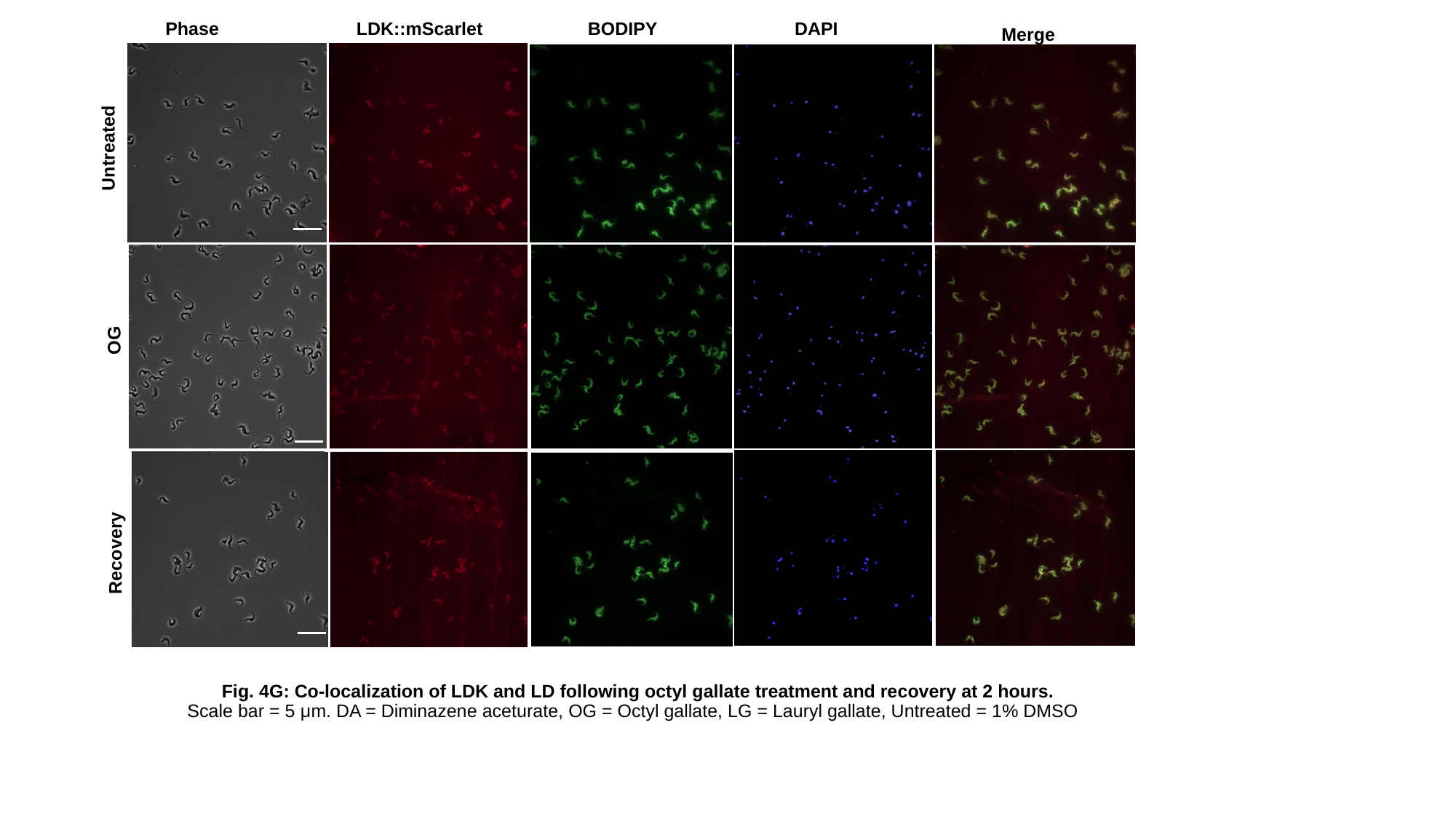

LDK::mScarlet
BODIPY
Phase
DAPI
Merge
Untreated
OG
Recovery
# Fig. 4G: Co-localization of LDK and LD following octyl gallate treatment and recovery at 2 hours. Scale bar = 5 μm. DA = Diminazene aceturate, OG = Octyl gallate, LG = Lauryl gallate, Untreated = 1% DMSO
